# Identification of cellular heterogeneity and key signaling pathways associated with vascular remodeling and calcification in young and old primate aortas based on single-cell analysis

**DOI:** 10.18632/aging.204442

**Published:** 2022-12-23

**Authors:** Yehu Yin, Congcong Huang, Zidi Wang, Pan Huang, Shanshan Qin

**Affiliations:** 1 Department of Stomatology, Taihe Hospital and Hubei Key Laboratory of Embryonic Stem Cell Research, School of Basic Medical Sciences, Hubei University of Medicine, Shiyan 442000, Hubei, P.R. China; 2 Institute of Medicine, Jishou University, Jishou 416000, P.R. China; 3 Laboratory of Tumor Biology, Academy of Bio-Medicine Research, Hubei University of Medicine, Shiyan 442000, Hubei, P.R. China

**Keywords:** vascular aging, scRNA-seq, cellular heterogeneity, cell-cell communication, EGF

## Abstract

Aging of the vascular system is the main cause of many cardiovascular diseases. The structure and function of the blood vessel wall change with aging. To prevent age-related cardiovascular diseases, it is essential to understand the cellular heterogeneity of vascular wall and changes of cellular communication among cell subpopulations during aging. Here, using published single-cell RNA sequencing datasets of young and old monkey aortas, we analyzed the heterogeneity of vascular endothelial cells and smooth muscle cells in detail and identified a distinct endothelial cell subpopulation that involved in vascular remodeling and calcification. Moreover, cellular communication that changed with aging was analyzed and we identified a number of signaling pathways that associated with vascular aging. We found that EGF signaling pathway play an essential role in vascular remodeling and calcification of aged aortas. This work provided a better understanding of vascular aging and laid the foundation for prevention of age-related vascular pathologies.

## INTRODUCTION

Cardiovascular disease is a leading cause of death worldwide and aging of the vasculature plays a central role in the development of cardiovascular disease [1]. Many age-related cardiovascular diseases are because of structural alterations of the vascular system during aging, including vascular wall thickening, collagen deposition, perivascular fibrosis and vascular calcification [2]. In the healthy artery, different cell types and layers of the vessel wall can change its structure and function according to extravascular environment or hemodynamic demands to maintain blood pressure homeostasis [3]. With advanced aging, the large arteries dilate, and their walls become thicker and stiffer due to collagen and calcium deposition and fragmentation of the elastic fibers. These changes can increase the stiffness of arteries [4]. Large-artery stiffness has profound consequences for cardiovascular health and is likely responsible for a large proportion of the global chronic cardiovascular disease burden [5].

The arterial vascular wall is mainly composed of endothelial cells (ECs), smooth muscle cells (SMCs), adventitial fibroblasts (AFs) and extracellular matrix (ECM) [6]. ECs are in the internal surface of vasculature and play a crucial role in sensing hemodynamic changes and maintaining vascular homeostasis. Various information is transmitted from ECs to other cells comprising the arteries [7]. In addition, ECs can produce cytokines and growth factors to induce SMCs phenotype switching from contractile to a more synthetic phenotype, resulting in stiffened vasculature [8]. The SMCs reside in the medial layer of major blood vessels and connect to a fenestrated network of elastin and collagen fibers. It maintains vessel tone through contraction and relaxation, and also plays a vital role in maintaining and remodeling the ECM of blood vessels [9]. SMCs are the most abundant cell type in the arterial vessel wall and they can regulate arterial remodeling by overproducing various ECM components to maintain vascular homeostasis [10]. In addition, in response to a variety of stimuli, SMCs can undergo osteogenic differentiation and mineralization, further driving vascular calcification [11]. The outermost layer of the vessel wall is comprised of AFs and ECM, maintaining structural integrity under peak mechanical load. All components of the vessel wall contribute to vascular remodeling and calcification during aging [12, 13]. Studying the communications between different cell types within vascular wall, such as ECs and SMCs, may contribute to better understand the cellular mechanisms of vascular aging, and to discover specific therapeutic targets to halt the progression of arterial stiffness and calcification.

Single-cell RNA sequencing (scRNA-seq) is a powerful research tool that can systematically characterize cellular heterogeneity and lineage progression at the single cell level [14, 15]. It has been employed to investigate cellular components and molecular properties of different tissues and organs [16–19]. Recently, researchers performed scRNA-seq on lesion-prone aortas and coronary arteries in young and aged cynomolgus monkeys, and identified unique molecular signatures and cell type specific changes of aged primate vasculature at single cell level [20]. Moreover, morphologically, senile aortas exhibited increased wall thickness and arterial calcification, which indicated that vascular remodeling has occurred in aged monkey vasculature during aging. Although the molecular characteristics of the aged monkey vasculature has been studied, the features of each subpopulation and the changes of cell-cell communication between different cell types within aged vasculature are still not clear. CellChat is an analytic tool that can be used to analyze intercellular communication networks from scRNA-seq data [21]. It has been successfully employed to investigate the intercellular communication status of healthy and diseased aortas [22]. Here, we used CellChat to deduce the cellular communication of age vasculature with published datasets. Our work inferred the intercellular communication status of young and old aortas, and predicted potential signaling pathways altered with aging, which paves the way for new therapies against human cardiovascular diseases.

## RESULTS

### Single-cell profile of vascular wall cells

The arterial vascular wall is mainly composed of ECs, SMCs, AFs and ECM [6]. All components of the vessel wall interact with each other to maintain the physiological homeostasis of blood vessels. In the healthy artery, the vessel wall can change its structure and function to adapt extravascular environment or hemodynamic demands [3]. However, with the aging and function losing of vasculature, these adaptive changes do not return to baseline level but instead initiate pathological vascular remodeling, which further leads to the development of cardiovascular disease [3]. These vascular remodeling was obviously observed in senile aortas, represented as increased wall thickness, fibrous cap formation, arterial calcification and fragmentation of the elastic lamina [20].

**Figure 1:**
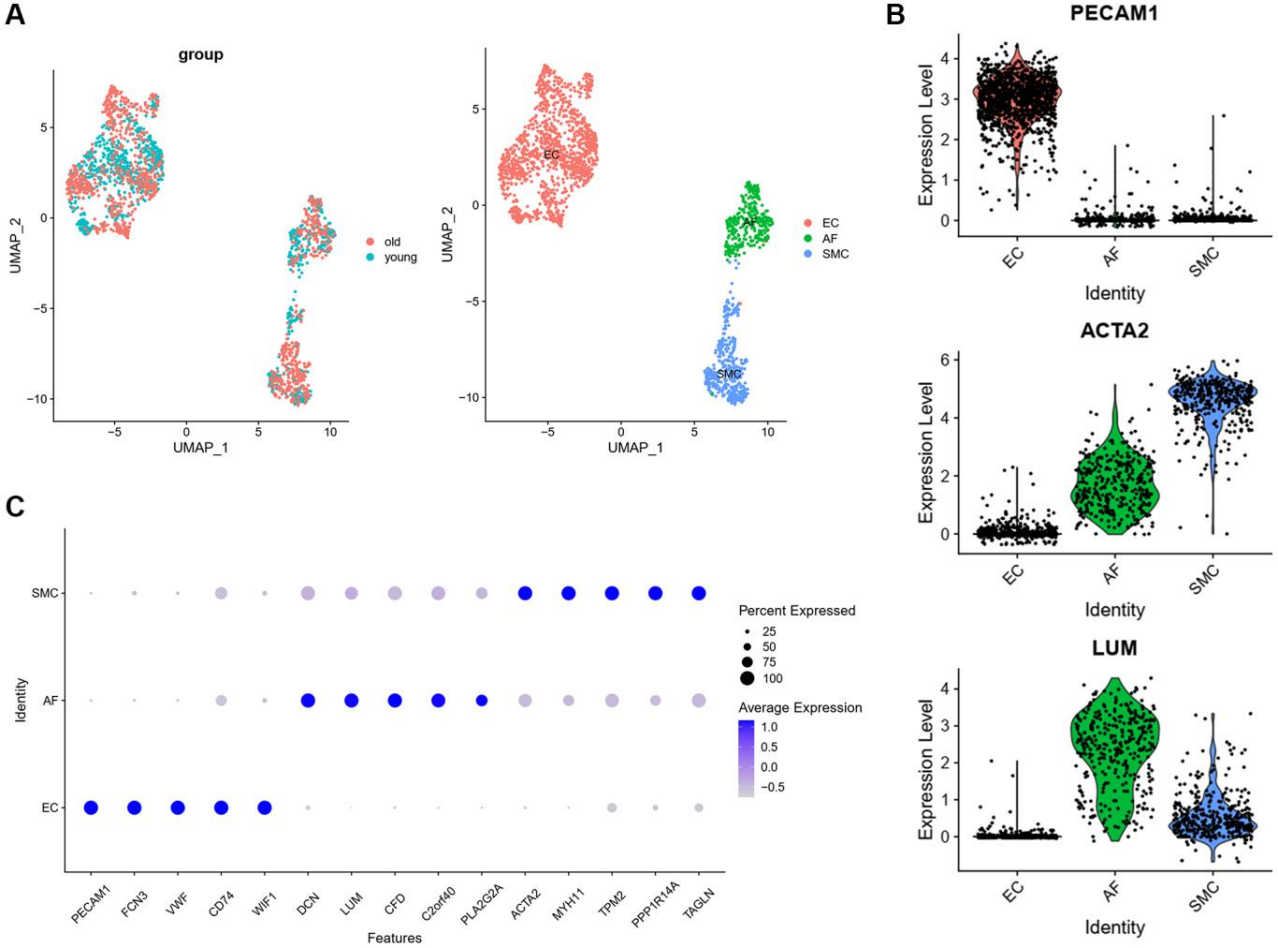
**Transcriptomic profile of aortic arches from young and old monkeys.** (**A**) UMAP plots of the scRNA-seq datasets displayed by group and cell type. (**B**) The expression of classic markers in each cell type. (**C**) Top 5 marker genes in each cell type. Abbreviation: UMAP: Uniform Manifold Approximation and Projection.

In order to analyze the cellular heterogeneity and the changes of cellular communication between different cell types of aged monkey vasculature. We analyzed a published scRNA-seq dataset of aortic arches from four young (age, 4–6 years) and four old (age, 18–21 years) male cynomolgus monkeys (GEO accession nr. GSE117715). As we focused on cells forming the wall of blood vessel, we only extracted ECs, SMCs and AFs from cell types that were identified in the original studies. The cells of ECs, SMCs and AFs were merged into an integrated Seurat object. After data preprocessing and dimensional reduction, ECs, SMCs and AFs were separately displayed in the UMAP plot (Figure 1A). The traditional marker genes of ECs (PECAM1), SMCs (ACTA2) and AFs (LUM) were specifically expressed in ECs, SMCs and AFs respectively (Figure 1B), which indicated that the definition of these cell types was correct. To further verify it, we identified the top 5 highly expressed genes in each cell type, and found that those genes were all cell type specific marker genes (Figure 1C). These results further verified the correctness of cell types.

### EC1 may be a subpopulation related to arterial remodeling and vascular calcification

**Figure 2:**
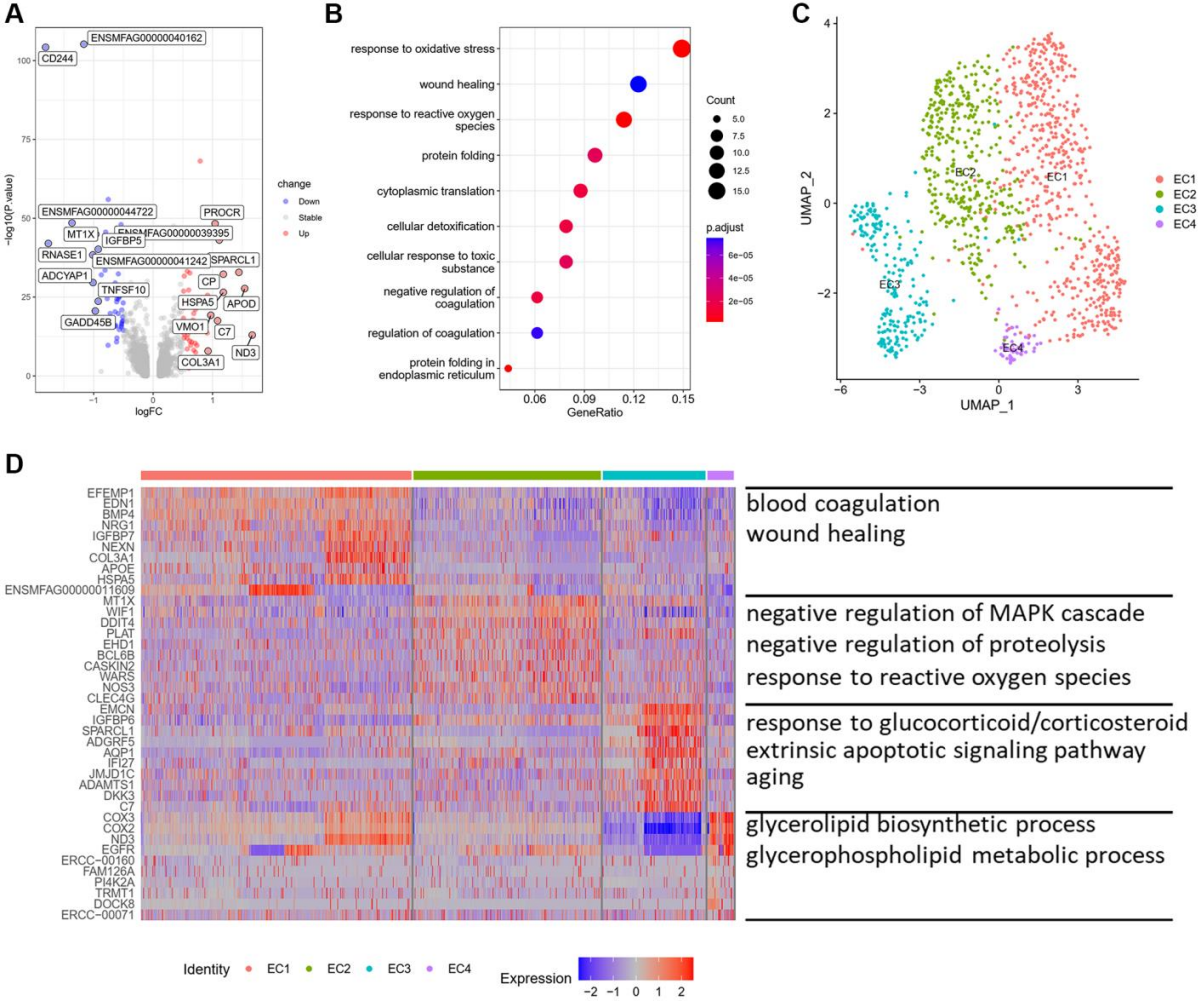
**Characteristics of ECs and their subpopulations.** (**A**) Volcano plot of DEG (average log fold change > 0.25, *P* value < 0.01) between young and old group. (**B**) GOBP analysis of upregulated DEG. (**C**) UMAP plots of EC subpopulations displayed by cell type. (**D**) Heatmap showing the top 10 genes of each EC subpopulation (left) and GOBP enriched by upregulated DEG in each subpopulation (right). Abbreviations: DEG: differentially expressed genes; GOBP: gene ontology biological process.

ECs, a single cell layer in the internal surface of the vasculature, play a crucial role in sensing hemodynamic changes and maintaining vascular homeostasis. To figure out the effect of aging on ECs of monkey vasculature, we performed differentially expressed genes (DEGs) analysis between old and young aortic arteries, and found 162 upregulated genes and 223 downregulated genes (Figure 2A). Most of these upregulated genes were associated with extracellular matrix formation, such as COL3A1, FN1 and BGN. Moreover, genes related to lipid metabolism and transport, including APOD and APOE, were highly expressed in old aortic arteries. Functional enrichment analysis showed that these upregulated genes were mainly enriched in biological processes of response to oxidative stress, wound healing and protein folding (Figure 2B). These results indicated that the function and structure of ECs may have changed during aging.

To further analyze the structural and functional heterogeneity of ECs, we performed unsupervised clustering to ECs alone and found out four distinct subpopulations (Figure 2C). We then identified the top 10 highly expressed genes of each subpopulation. Each of the four ECs subsets expressed distinct marker genes, suggesting the presence of discrete subpopulations (Figure 2D). We observed that EC1 highly expressed genes related to extracellular matrix organization and ossification, such as FN1, COL3A1, BMP4 and SPARC (Supplementary Figure 1). The biological processes of EC1 were mainly enriched in blood coagulation, wound healing and response to transforming growth factor beta, which are involved in ECM remodeling and vascular fibrosis (Figure 2D). While, the biological processes of EC2 subpopulation were related to negative regulation of MAPK cascade, negative regulation of proteolysis and response to reactive oxygen species. The upregulated genes in EC3 were enriched in biological processes of response to glucocorticoid/corticosteroid, extrinsic apoptotic signaling pathway and aging (Figure 2D). EC4 was enriched for glycerolipid biosynthetic process and glycerophospholipid metabolic process. These results showed that ECs had structural and functional heterogeneity and EC1 may be a subpopulation related to arterial remodeling and vascular calcification.

### The structural and functional heterogeneity of SMCs

**Figure 3:**
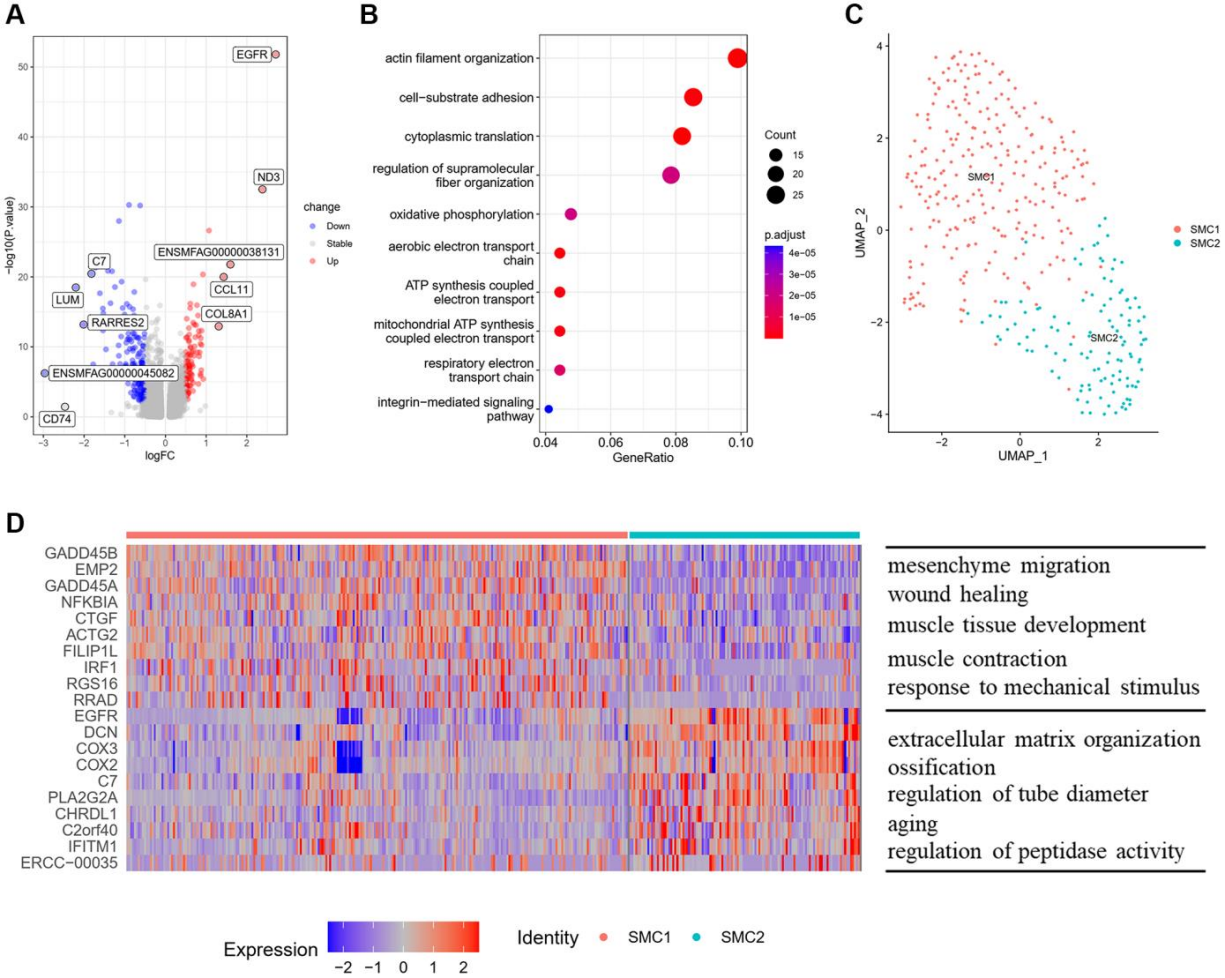
**Characteristics of SMCs and their subpopulations.** (**A**) Volcano plot of DEG (average log fold change > 0.25, *P* value < 0.01) between young and old group. (**B**) GOBP analysis of upregulated DEG. (**C**) UMAP plots of SMC subpopulations displayed by cell type. (**D**) Heatmap showing the top 10 genes of each SMC subpopulation (left) and GOBP enriched by upregulated DEG in each subpopulation (right). Abbreviations: DEG: differentially expressed genes; GOBP: gene ontology biological process.

The SMCs reside in the medial layer of major blood vessels. They play a vital role in vascular remodeling and calcification [9]. To figure out the effect of aging on SMCs of monkey vasculature, we performed DEG analysis between old and young aortic arteries, and found 573 upregulated genes and 816 downregulated genes (Figure 3A). These upregulated genes in old aortic arteries were mainly enriched in biological processes of actin filament organization, cell−substrate adhesion and cytoplasmic translation (Figure 3B).

To further analyze the structural and functional heterogeneity of SMCs, we performed unsupervised clustering to SMCs and identified two different subpopulations (Figure 3C). We then identified the top 10 highly expressed genes, and performed functional enrichment analysis for these upregulated genes of each subpopulation (Figure 3D). SMC1 had a contractile phenotype and highly expressed traditional marker genes of SMCs, such as ACTA2 and CNN1 (Supplementary Figure 2). The biological processes enriched by the upregulated genes in SMC1 were involved in muscle tissue development and response to mechanical stimulus, which are required for maintaining normal function of arteries (Figure 3D). While, SMC2 seemed to have a synthetic VSMC phenotype, with losing contractility markers and expressing proteins involved in extracellular matrix organization (Supplementary Figure 2). Besides, SMC2 highly expressed ossification related genes (EGFR, CHRDL1) and aging-related genes (SERPINF1, APOE), and the biological processes of these upregulated genes were enriched in ossification and aging.

### Deciphering cell-cell communication in all cell subpopulations of vascular wall

**Figure 4:**
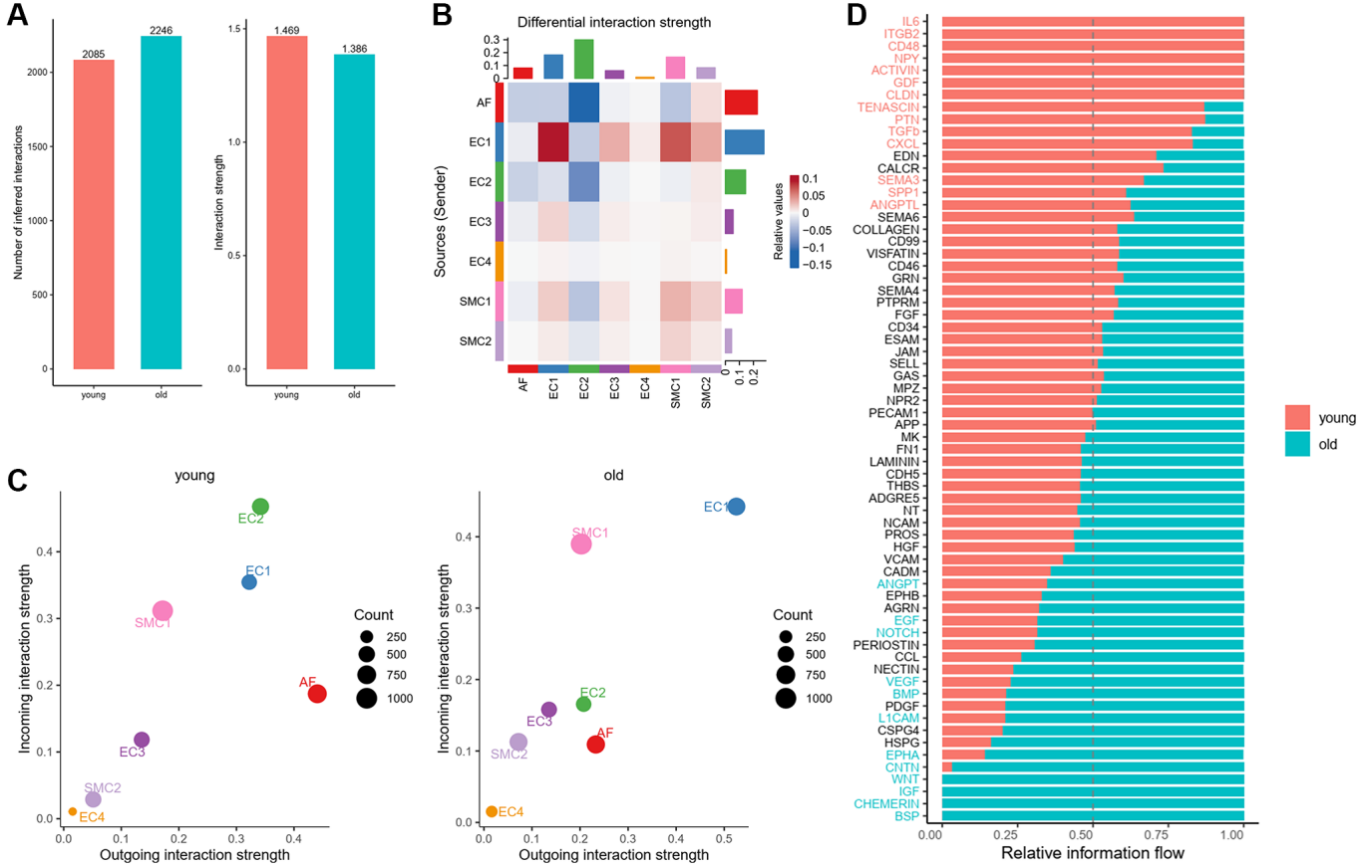
**Overview of intercellular communication between young and old group.** (**A**) Total interaction number and strength in young and old group. (**B**) Heatmap of differential interaction strength in young and old group. (**C**) Scatter plot of incoming and outgoing interaction strength of each cell population in young and old group. (**D**) Overall information flow of each signaling pathway in young and old group.

Intercellular communication between different cell types of vascular wall is important for aging process and aging related arterial remodeling [23]. In order to distinguish the changes of cellular communication between different cell subpopulations of aged aortas, using CellChat, we evaluated cell-cell communication patterns in seven subpopulations of aortic wall cells, including four EC subpopulations, two SMC subpopulations and one AF subpopulation. Results of CellChat analysis revealed 2,085 total ligand-receptor interactions in the young group and 2,246 interactions in the old group (Figure 4A). But the total interaction strength of the old group was moderately lower than that of the young group (Figure 4A).

To figure out the signal changes of each subpopulation, we compared the outgoing and incoming signals of each cell population in young and old aortas. We found that the signals from EC1 to SMC1 and SMC2 were increased significantly, while the signals received by AFs were all slightly decreased (Figure 4B). Compared with young group, the incoming signals of SMC1, SMC2 and EC1 were all strengthened, and that of EC2 were weakened obviously (Figure 4C). To identify specific signaling pathways that changed in the aged aortas, we compared the overall information flow for each signaling pathway between young and old group. Results showed that a number of signaling pathways in old group were stronger than that in young group, such as VEGF, BMP, NOTCH and EGF. Moreover, there were four signaling pathways (BSP, CHEMERIN, IGF, WNT) unique to old group (Figure 4D).

### The altered signals in subpopulations of aged aortas

**Figure 5:**
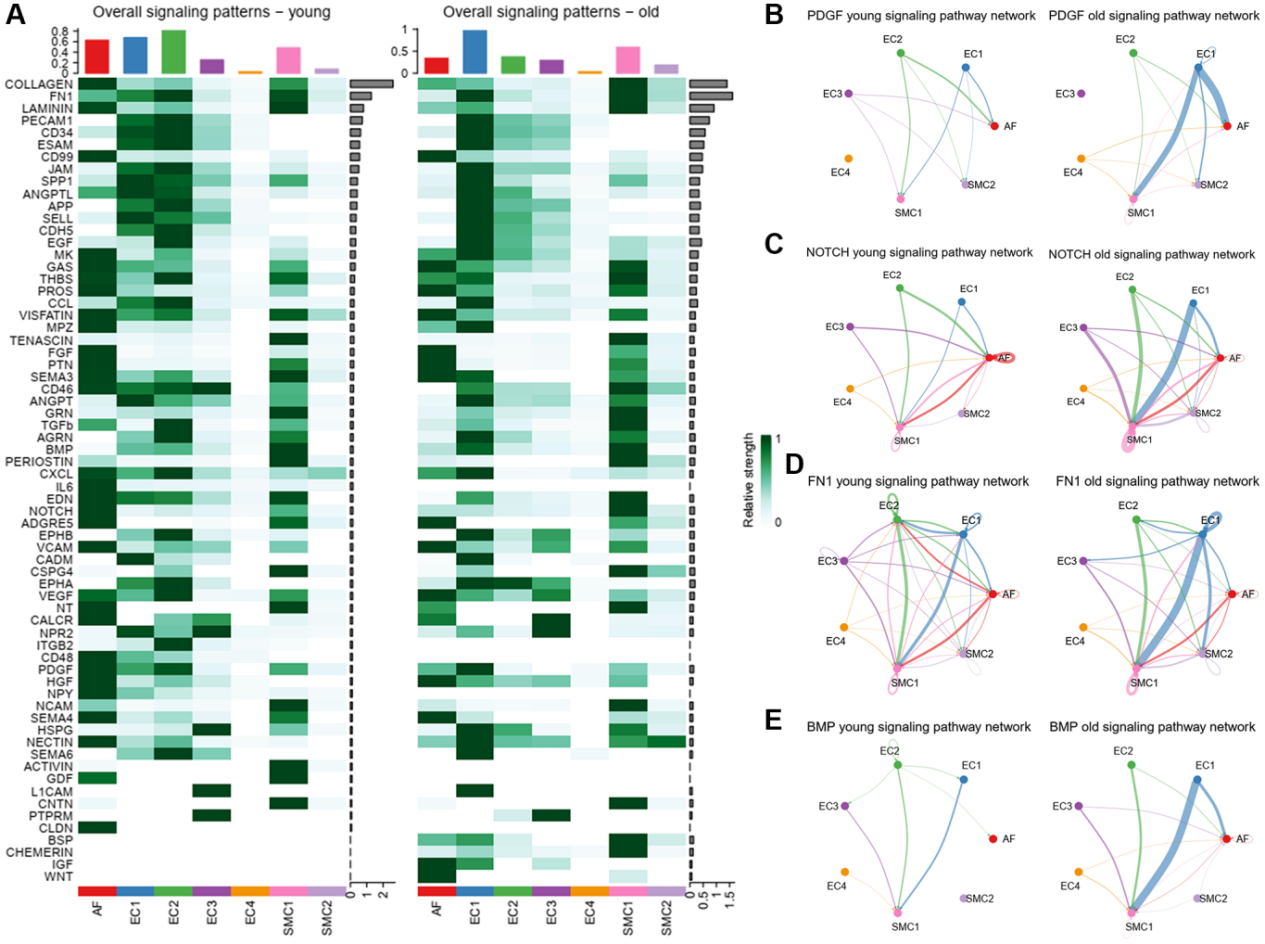
**Overall signaling patterns and inferred signaling pathways.** (**A**) Overall signal strength of each signaling pathway in each cell population. (**B**) Circle plot of PDGF signaling network in young and old group. (**C**) Circle plot of NOTCH signaling network in young and old group. (**D**) Circle plot of FN1 signaling network in young and old group. (**E**) Circle plot of BMP signaling network in young and old group.

To further analyze the alteration of signals in each cell subpopulation, we compared the signal strength of each signaling pathway between young and old group (Figure 5A). Most of the overall signaling pathways in AFs were weakened in old group, such as COLLAGEN and LAMININ (Supplementary Figure 3). However, we found that the VEGF signaling pathway was increased in aged aortas and it mainly sent from AFs to ECs (Supplementary Figure 3). VEGF was a vascular endothelial-derived growth factor, which can regulate multiple biological functions, including vascular permeability, angiogenesis and endochondral ossification [24]. In contrast, a number of signaling pathways were increased in EC1 subpopulation of aged aortas, such as PECAM1, ESAM and CDH5. PECAM1 is closely correlative with cell migration, proliferation, apoptosis, signal transduction and cellular immunity [25]. ESAM is involved in leukocyte extravasation and VEGF-induced vascular permeability [26]. Interestingly, these signaling pathways have similar changes in the aged aortas. The cell-cell communications between EC2 and EC3 were weakened, but the communications between EC1 and EC3 were strengthened in the aged aortas (Supplementary Figure 4). These results indicated that the cellular communications of ECs have changed during aging.

Similarly, a number of signaling pathways were increased in SMC subpopulations. For example, the ANGPT, THBS and CSPG4 were overall highly expressed signaling pathways in SMC1 (Supplementary Figure 5). ANGPT is a second endothelial cell specific ligand–receptor signaling system, which is involved in postnatal angiogenesis, vessel remodeling, vascular permeability and inflammation to maintain vascular homoeostasis in adult physiology [27]. Moreover, THBS are evolutionarily conserved, calcium-binding glycoproteins that contribute to wound healing and angiogenesis, vessel wall biology, connective tissue organization, and synaptogenesis [28]. Recent analysis found that THBS signaling pathway was associated with abdominal aortic aneurysm [22], which is a kind of aged related cardiovascular disease. These results indicated that the cellular communications of SMCs have changed during aging.

### The altered signals between EC1 and SMC

Adequate cooperation between ECs and SMCs is required to maintain vascular normal function. Disturbances in this balance predispose the vessel to initiate pathological change. As EC1 is a subpopulation related to vascular remodeling and calcification, we analyzed the changed signals that sent from EC1 to SMCs. PDGF is a platelet-derived growth factor, which plays a prominent role in the pathogenesis of cardiovascular disease [29]. We observed that increased PDGF signal was mainly sent from EC1 to SMC1 and AFs (Figure 5B). The NOTCH signaling pathway, which is involved in cell fate determination and tissue homeostasis [30], was also strengthened in the old group (Figure 5C). As we mention above, the aged aortas have phenotypes of vascular remodeling and calcification. We then examined the signaling pathway of FN1, which is associated with vascular remodeling and calcification. We found that the signaling pathway of FN1 was significantly increased from EC1 to SMC1 in the old group (Figure 5D). Bone morphogenetic proteins (BMPs) are a group of ligand proteins that involved in regulating vascular calcification and bone formation [31]. In our study, the BMP signaling pathway and its ligand-receptor pairs were all increased from EC1 to SMC1 in the old group (Figure 5E), implying its key role in vascular aging. These results indicated that these changed signals between EC1 and SMCs may relate to the arterial remodeling and calcification of age aortas.

**Figure 6:**
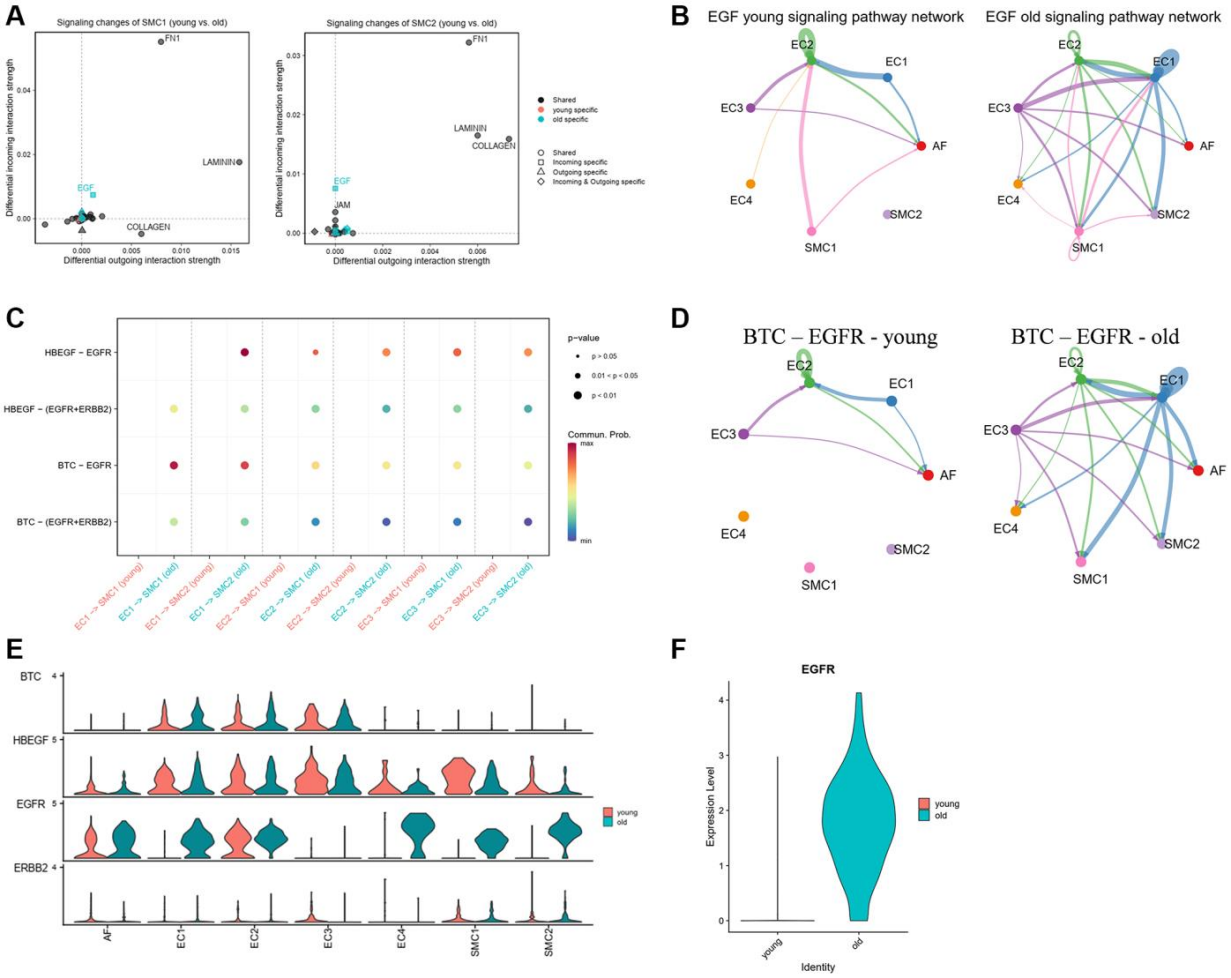
**EGF signaling in young and old group.** (**A**) Signaling changes of SMC1 and SMC2. (**B**) Circle plot of EGF signaling network in young and old group. (**C**) The communication probability of all the significant ligand-receptor pairs that contributed to EGF signaling. (**D**) Circle plot of BTC-EGFR ligand-receptor pairs in young and old group. (**E**) The expression of genes of EGF signaling pathway in each subpopulation and group. (**F**) The expression of EGFR in young and old group.

SMCs are the major cells in the vascular wall and play an important role in arterial remodeling and calcification. To further analyze the signaling changes in SMCs, we compared the incoming and outcoming signals between young and old groups. Results showed that FN1, LAMININ and COLLAGEN were the most changed signals in SMCs (Figure 6A). We noticed that EGF signaling pathway was specifically received by SMCs in the old group. This signal was mainly sent from EC3 to EC2 in the young group, but in the old group, it was changed and mainly sent to EC3, SMC1 and SMC2 (Figure 6B). Besides, the ligand-receptor pairs of EGF signaling pathway were all strengthened from ECs to SMCs in the old group (Figure 6C). Among these ligand-receptor pairs, BTC-EGFR ligand-receptor was increased significantly in the old group. EC1 and EC3 were the main output subpopulations of BTC-EGFR signaling, while the SMCs and AFs were the receiver of that (Figure 6D). To further verify it, we analyzed the gene expression of ligand-receptors of EGF signaling pathway in each subpopulation. We observed that the genes of ligands were highly expressed in ECs, while the receptors were mainly expressed in AFs and SMCs (Figure 6E). Notably, the expression of EGFR, an important receptor of EGF signaling pathway, was significantly higher in EC1, SMC1 and SMC2 of the old group than the young group (Figure 6E). Besides, the average expression of EGFR in old group was also higher than that in young group (Figure 6F). These results indicated that EGF signaling pathway may play an important role in vascular aging.

## DISCUSSION

Aging is one of the major risk factors associated with cardiovascular disease. With chronological aging, the arteries, especially the large arteries, undergo significant structural and functional changes [32]. These adaptive changes include vessel dilatation, vascular wall thickening, vascular fibrosis and calcification [33]. Age-related functional changes are the result of phenotypic alterations of different cell types, such as ECs, SMCs and AFs [13]. ECs are constantly exposed to stress such as mechanical and humoral factors, and play an important role in maintaining vascular physiological balance. Alterations of endothelial structure and function are closely related to aging of the cardiovascular system. Recent advances in single-cell RNA-sequencing make it possible to identify and characterize cellular subpopulations and a lot of studies have shown that endothelial cells have cellular heterogeneity [34, 35]. In our study, we identified four EC subpopulations. Besides the normal functional EC2 subpopulation and lipid metabolism related EC4 subpopulation, we found one distinct EC subpopulation (EC1). EC1 highly expressed genes related to ossification, such as BMP4 and SPARC. SPARC is a cysteine-rich acidic matrix-associated protein, and it can bind collagen and hydroxyapatite crystals enhancing mineralization of the collagen matrix in bones [36]. Moreover, a number of fibrosis related genes were highly expressed in EC1 subpopulation. COL3A1 encoded the pro-alpha1 chains of type III collagen, a fibrillar collagen that functions in cell adhesion, migration, proliferation and differentiation [37]. Ctgf, connective tissue growth factor, was a central mediator of tissue remodeling and fibrosis, and involved in the control of cell proliferation, differentiation, adhesion and angiogenesis [38]. Besides, FN1, BGN and COL8A1 were also highly expressed in EC1 subpopulation. These results indicated that EC1 was a distinct EC subpopulation that related to vascular fibrosis and calcification. Cell-cell communication analysis found that signaling pathways of PECAM1, ESAM and CDH5 were strengthened in EC1 of aged aortas. These signals mainly related to cell migration and vascular permeability, which indicate that the endothelial permeability and function has changed during aging.

The arterial wall is made up of three distinct layers and the tunica intima is originally constituted by a single layer of ECs. The thickness of the arterial wall mainly occurs in the intimal layer and the intimal thickness significantly increased with aging in rabbits and primates [20, 39]. The diffusely thickened aging intima mainly consists of matric proteins, collagen, glycosaminoglycans, and SMCs that are thought to have migrated from the media [40]. Indeed, we found that genes associated with cell migration, such as EMP2 and MACF1, were highly expressed in SMC1. Moreover, the upregulated genes of SMC1 were enriched in the biological pathways of tissue migration and mesenchyme migration, which further indicated the migration of SMCs to intima. In addition, age-associated changes also occur in the medial layer of the central arteries and the medial layer of aged aortas exhibits an increased collagen content and frayed elastin [41]. SMCs in the medial layer of the vessel wall play a key role in maintaining vessel structure and function [42]. A variety of structural and functional changes occur in SMCs with aging. In normal arteries, SMCs have a contractile phenotype, and perform the normal function of blood vessels. However, under pathological conditions, such as aging and injury, SMCs can switch their contractile phenotype to synthetic phenotype, represented by down-regulating contractile proteins and remodeling the ECM to adapt the local physiological conditions [43]. In our study, we identified two SMC subpopulations. SMC1 highly expressed muscle contraction related genes and seemed to have contractile phenotype, while SMC2 had the synthetic phenotype and downregulated the contractile proteins ACTA2 and CNN1. Moreover, SMC2 highly expressed genes of extracellular matrix organization, such as COL4A3, DCN and LUM. These results indicated that age-related remodeling of aortas is associated with smooth muscle phenotypic transformation and extracellular matrix remodeling.

Vascular wall cells are mainly composed of ECs, SMCs and AFs and they work together to maintain the normal physiological function of blood vessels. Within a multicellular environment, cell-cell interactions play a fundamental role in orchestrating organismal development, homeostasis and single-cell functions [44]. The expansion of protein–protein interaction databases and recent advances in RNA sequencing technologies have provided an opportunity to study the complex communication networks between cells in multicellular communities [44]. In this study, we analyzed the intercellular communication of vascular wall cells between the young and old aortas, especially focusing on communications between ECs and SMCs. We identified four distinct signaling pathways (BSP, CHEMERIN, IGF, WNT) that unique to old group (Figure 4D). BSP is a mineralized tissue-specific noncollagenous protein that usually deposit into the extracellular matrix of bone. It has the ability to nucleate hydroxyapatite crystal formation, and play a potential role in the initial mineralization of bone, dentin and cementum [45]. The BSP signaling mainly sent from EC1 to the SMC and AF (Supplementary Figure 6), and may involve in the arterial remodel and calcification of aged monkey aortas. CHEMERIN is an adipokine highly expressed in adipose tissue, which is association with inflammation, endothelial dysfunction, metabolic disorder, aberrant angiogenesis, VSMC proliferation and calcification [46]. Similarly, the CHEMERIN signaling was also sent from EC1, but it only received by SMC1 (Supplementary Figure 6), and may also play an important role in arterial remodel and calcification. Besides, WNT is a highly conserved signaling in the aberrant wound repair and fibrogenesis and plays important roles in cell fate determination, proliferation and cell polarity establishment [47, 48]. The WNT signaling was sent from AF to other cell types (Supplementary Figure 6), and may be related to vascular fibrosis and stiffness. These results indicated that the cellular communication between different cell types has changed in aged aortas, and these changed signaling pathways may be related to vascular remodeling and calcification of aged aortas.

Compared to the young group, the signals in EC1, SMC1 and SMC2 subpopulations were all strengthened significantly in the old group (Figure 4B, 4C). EC1 was a subpopulation related to arterial remodeling and vascular calcification, which highly expressed genes related to extracellular matrix organization and ossification. We found that the PDGF signaling pathway was increased in EC1 of aged aortas (Figure 5B). PDGF is an important signaling molecule in the initial phase of SMC differentiation and is known to induce SMCs differentiation towards a synthetic phenotype [49]. The PDGF signaling pathway was mainly sent from EC1 to SMC1 and may be involved in the phenotypic transformation of SMCs. Moreover, calcification related signals, such as FN1, NOTCH and BMP, were also sent from EC1, and received by SMC1. SMC2 seems to be an ossification and aging related SMC subpopulation. But the signals sent from EC1 were almost received by SMC1 rather than SMC2. The reason maybe that these signals mainly acted on SMC1 to promote it transdifferentiation, while SMC2 was a fully differentiated subpopulation.

In SMCs, we found that FN1, LAMININ and COLLAGEN were the most changed signaling pathways of SMCs in aged aortas and the communication between EC1 and SMCs of these signals all strengthened in the old group. Besides, we found that EGF signaling pathway was exclusively received by SMCs in aged aortas, which indicates its important role in vascular aging. EGF encodes a member of the epidermal growth factor superfamily that plays an important role in the growth, proliferation and differentiation of numerous cell types [50]. Moreover, EGF signaling has been reported to play an important role in endochondral ossification and bone homeostasis [51], which indicates that EGF signaling pathway may be involved in vascular calcification of aged aortas. Various ligands activate EGF signaling pathway, such as epidermal growth factor (EGF), betacellulin (BTC) and heparin-binding EGF-like growth factor (HBEGF). Binding of these ligands induces EGF receptor dimerization, and consequently activates tyrosine autophosphorylation and multiple downstream effectors [52]. In our study, we found that the contribution of BTC-EGFR ligand-receptor pair to EGF signaling pathway was increased significantly in aged aortas (Supplementary Figure 7). BTC is a dual-specificity ligand that binds to and activates EGFR. Overexpression of BTC results in an EGFR-dependent upregulation of cortical bone mass in the appendicular skeleton of mice [53], implying its role in vascular calcification. The signals of BTC-EGFR that sent from EC1 to SMCs were also strengthened in the aged aortas (Figure 6D). These results further indicated that EGF signaling pathway may play an important role in arterial remodeling and calcification during aging.

In conclusion, our analysis identified cellular heterogeneity of vascular wall cells and inferred some key intercellular signaling pathways that related to vascular remodeling and calcification of aged aortas. This work provided a better understanding of vascular aging and laid the foundation for prevention of age-related vascular pathologies.

## MATERIALS AND METHODS

### Data acquisition and preprocessing

We downloaded the integrated dataset provided in the NCBI GEO data repository (GEO accession no. GSE117715), which is a merged dataset containing expression matrix of all samples from eight young (4–6 years old) and eight old (18–21 years old) monkeys (*Macaca fascicularis*) [20]. To avoid the effect of sex-dependent factors on the composition of vascular cells, we only extracted the aortic dataset of 4 young and 4 old male monkeys. Since we focused on studying the changes in the structure and function of blood vessel walls with aging, only vascular component cells (ECs, SMCs and AFs) were selected for subsequent analysis. The R package Seurat (version 4.1.0) was used for subsequent analysis in R (version 4.1.2) environment [54]. To exclude poor-quality cells, cells that gene count per cell <1000 and >6500, and total counts >300000 were discarded. After quality control, data normalization and scaling were performed with Seurat package. The top 3000 variable genes for all samples were used for principal component analysis (PCA).

### Clustering of scRNA-seq data

The cluster of ECs and SMCs were performed with Seurat respectively. First, the extracted data of ECs or SMCs was normalized and scaled according to the Seurat manual. Similarly, the top 3000 variable genes were used for PCA, and uniform manifold approximation and projection (UMAP) was used for nonlinear dimensional reduction. The top 20 principal components were then used to cluster cells at the resolution of 0.3 for ECs and SMCs.

### Differential gene analysis and functional annotation

Cell type-specific and subcluster-specific markers of ECs or SMCs were analyzed using the “FindAllMarkers” function of Seurat with the Wilcox test. Differentially expressed genes (DEGs) between indicated cell types or sub-clusters were found with “FindMarkers” function, and genes with adjusted *p* values < 0.01 and |logFC| > 0.25 were considered to be DEGs [55]. Gene set enrichment analysis of the DEGs was performed using the R package clusterProfiler (version 4.2.2). GO terms with corrected *P* < 0.05 were considered significantly enriched.

### Analysis of cell–cell communication

Cell–cell communication between each cell subpopulation of vascular wall was performed using CellChat (version 1.1.3) with default settings [21]. Briefly, cells of ECs, SMCs and AFs were merged into an integrated Seurat object, and then subjected to CellChat pipeline to analyze. The ligand-receptor interaction database CellChatDB in human was used for following analysis, and the “computeCommunProb” function from CellChat was used to compute the communication probability. The signaling pathways were visualized with Hierarchy plot, Circle plot or Chord diagram, and the communication patterns of target cells were visualized with dot plots.

### Statistics

Statistical analysis was performed with default method in Seurat package. As we previously described [56–59], the *P* values for gene expression analysis in different groups were estimated using Wilcoxon Rank Sum test. As for cell communication analysis, statistical analysis was also performed with default method in CellChat package, and default method of “triMean” was used for computing the average gene expression per cell group. *P*-value < 0.05 was used for determining significant interaction.

### Data availability statement

The data that support the findings of this study are available in GEO database at (https://www.ncbi.nlm.nih.gov/geo/), reference number (GSE117715). Further inquiries can be directed to the corresponding authors.

## Supplementary Materials

Supplementary Figures
