# Supplementary material for: Identification of cellular heterogeneity and key signaling pathways associated with vascular remodeling and calcification in young and old primate aortas based on single-cell analysis
Source: Aging (Albany NY). 2022 Dec 23;15(4):982–1003. doi: 10.18632/aging.204442 (PMC10008505; doi:10.18632/aging.204442)
Supplement: Supplementary Figures [file aging-15-204442-s001.pdf]

SUPPLEMENTARY FIGURES

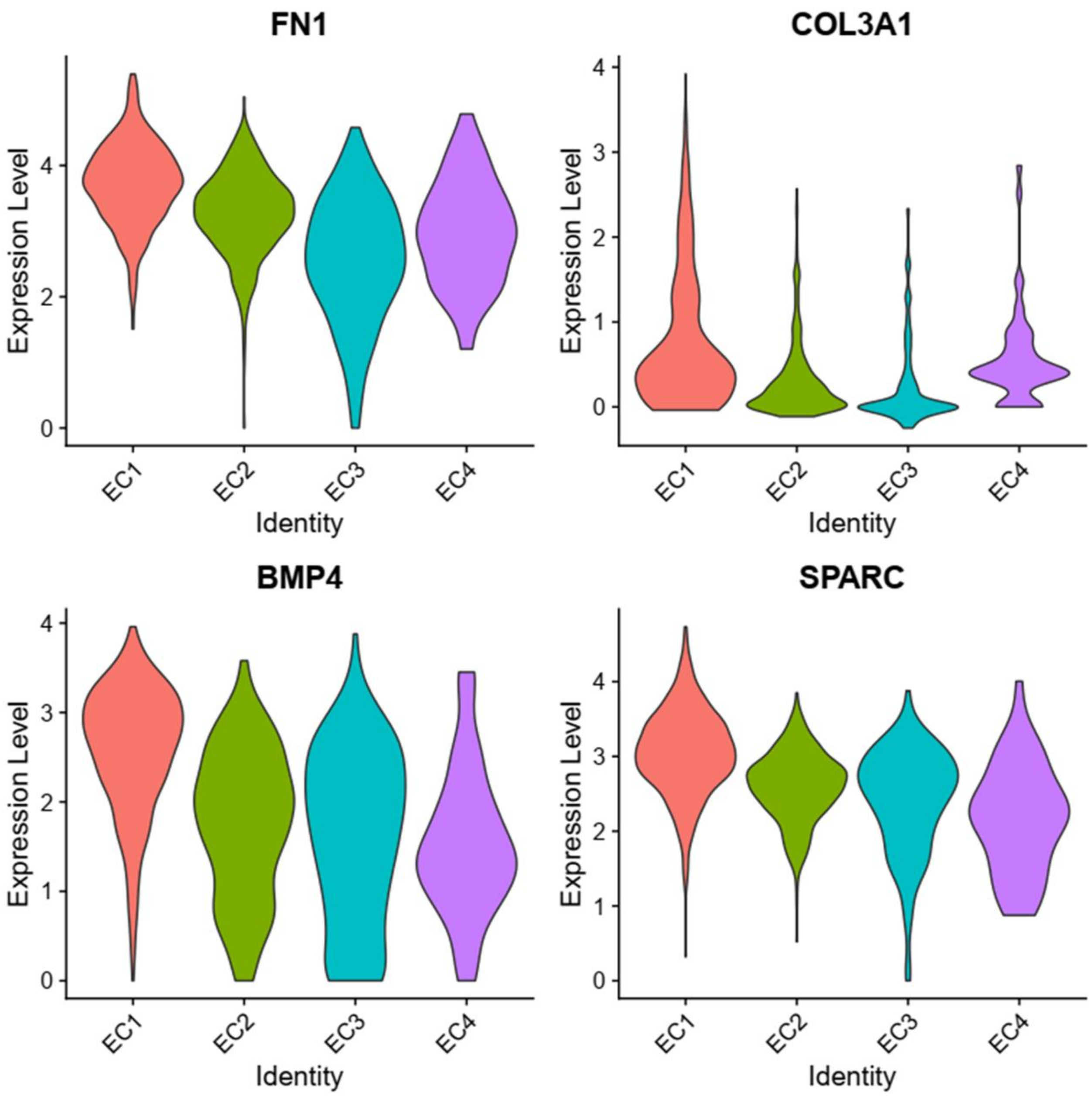

Supplementary Figure 1. The expression of FN1, COL3A1, BMP4 and SPARC in EC subpopulations.

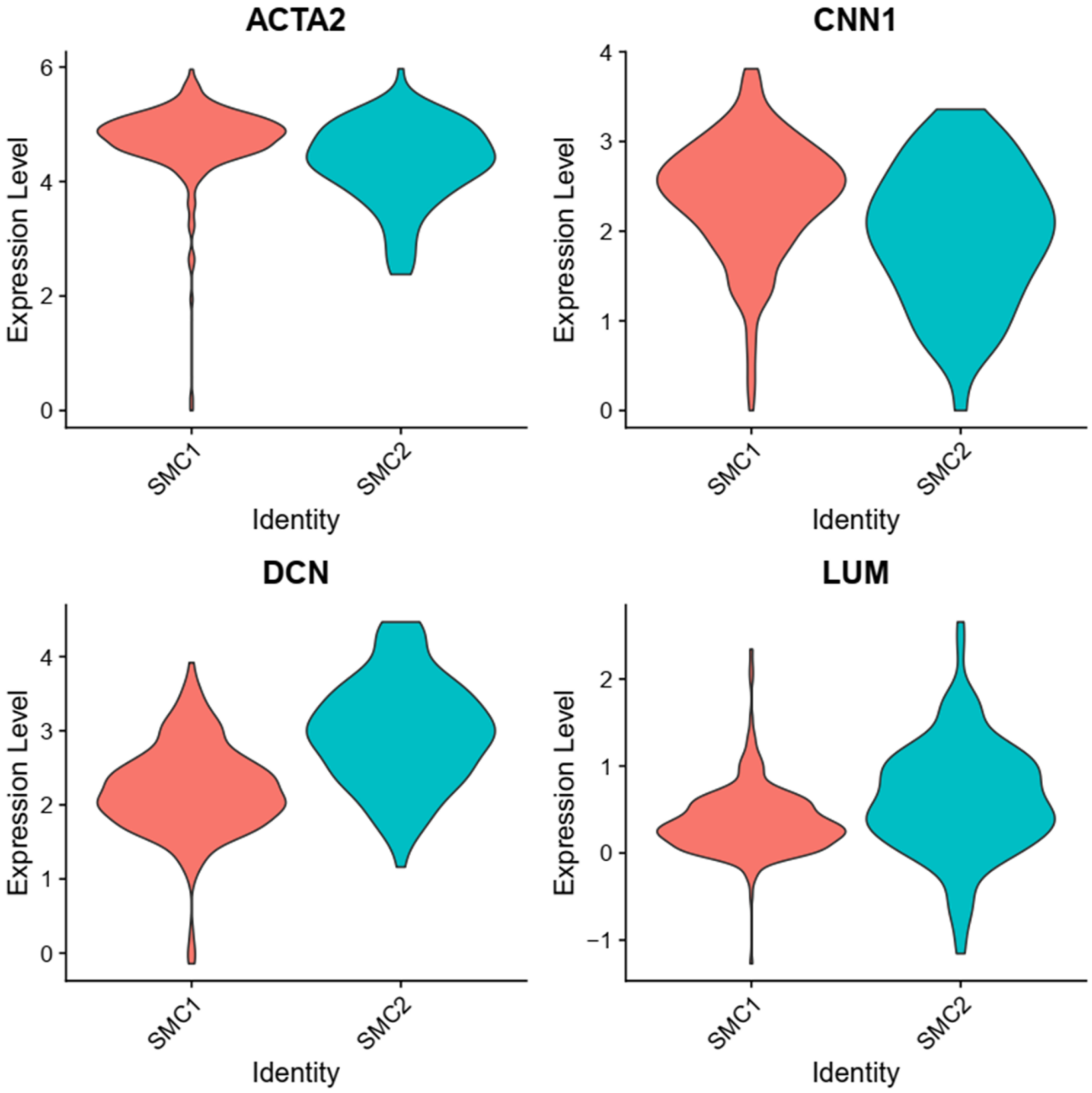

Supplementary Figure 2. The expression of ACTA2, CNN1, DCN and LUM in SMC subpopulations.

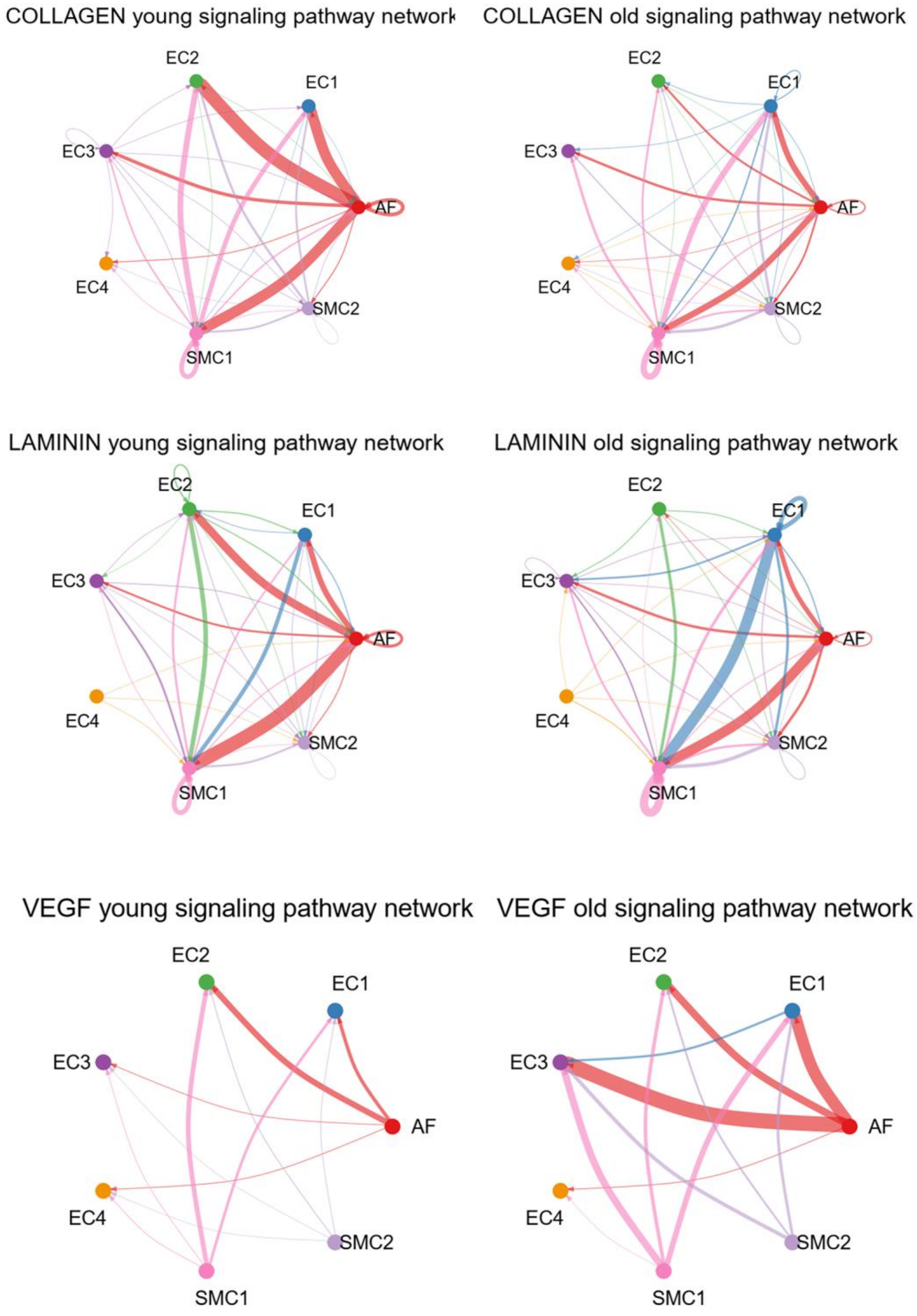

Supplementary Figure 3. Circle plot of signaling pathways that altered in AFs.

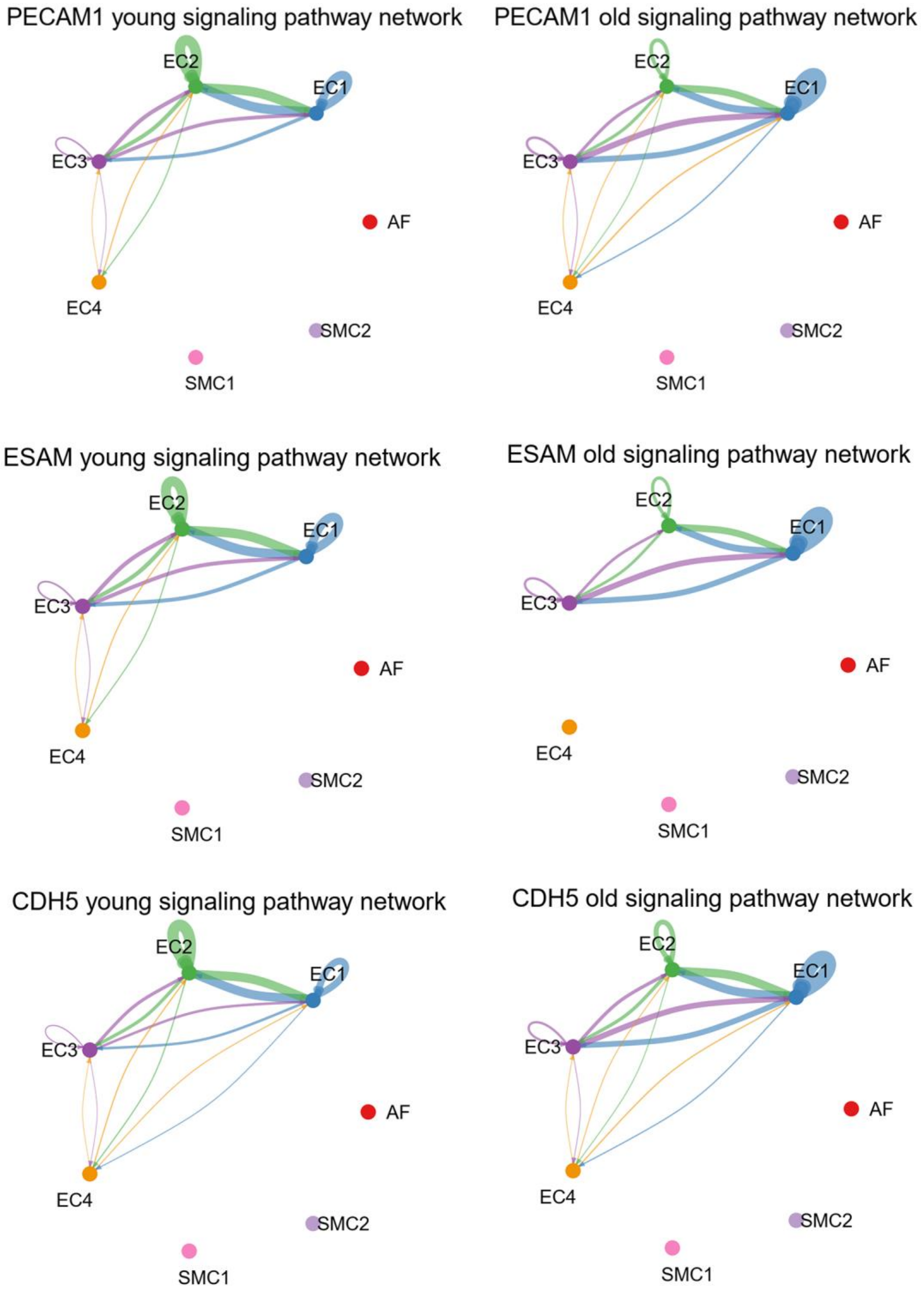

Supplementary Figure 4. Circle plot of signaling pathways that altered in ECs.

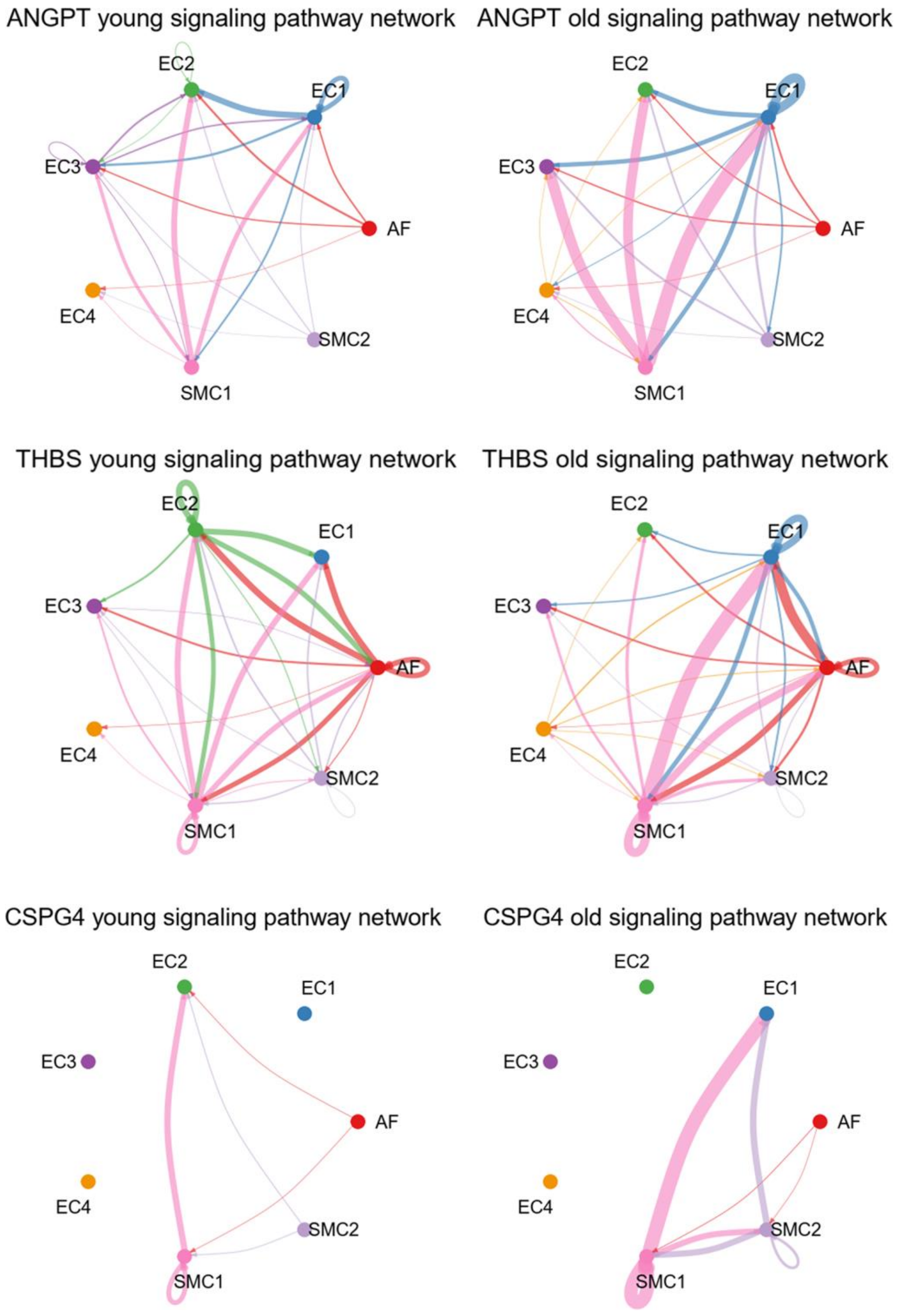

Supplementary Figure 5. Circle plot of signaling pathways that altered in SMCs.

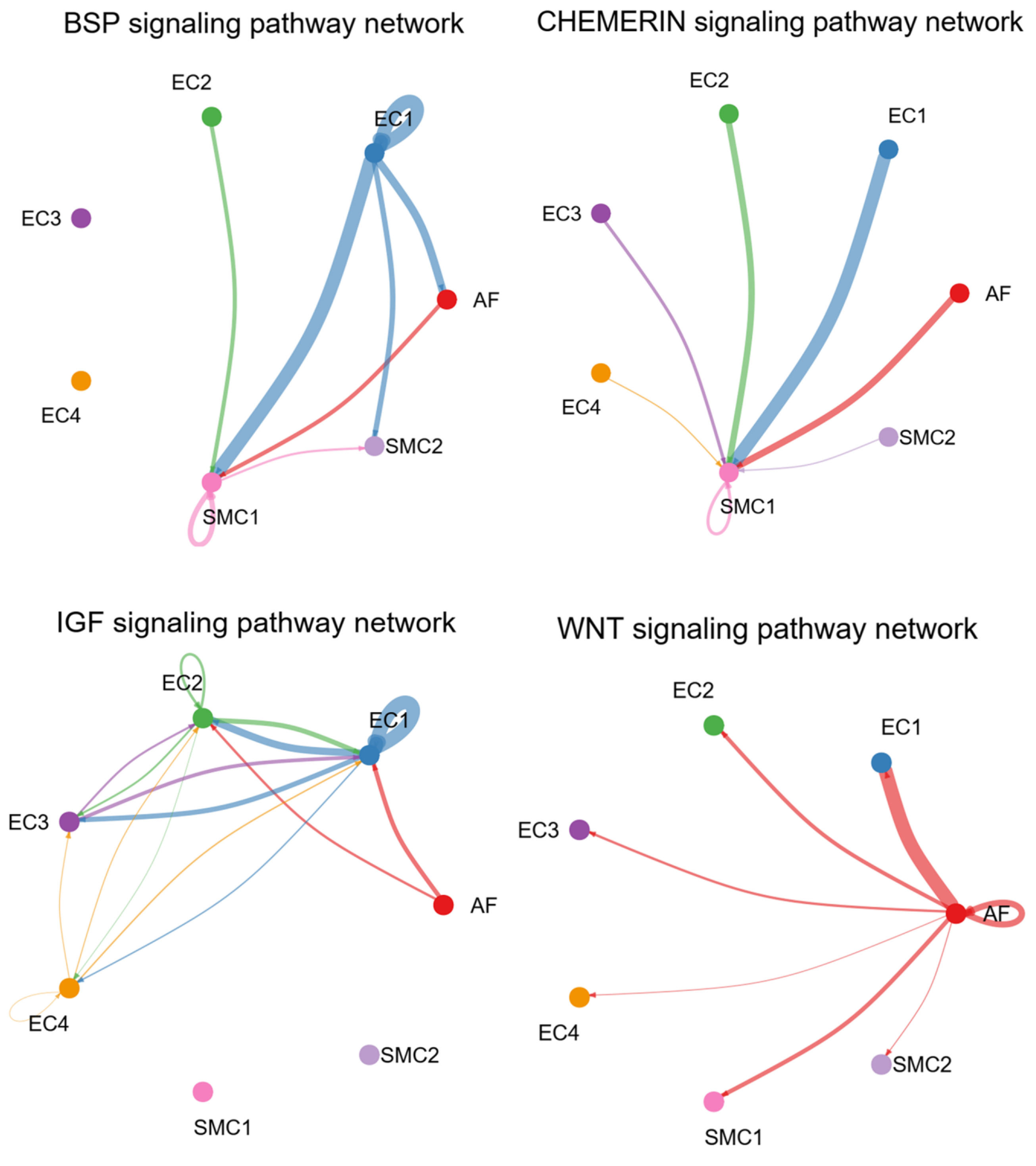

Supplementary Figure 6. Circle plot of signaling pathways that are unique to old group.

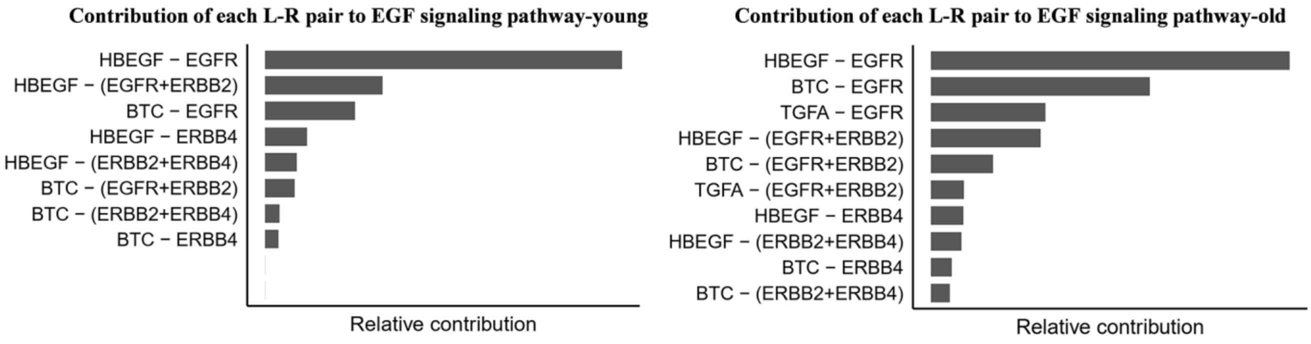

Supplementary Figure 7. Contribution of each L-R pair to EGF signaling pathway in young and old group.
